# Plasma Tie2 trajectories identify vascular response criteria for VEGF inhibitors across advanced biliary tract, colorectal and ovarian cancers

**DOI:** 10.1016/j.esmoop.2022.100417

**Published:** 2022-03-10

**Authors:** C. Zhou, J. O’Connor, A. Backen, J.W. Valle, J. Bridgewater, C. Dive, G.C. Jayson

**Affiliations:** 1 CRUK Manchester Institute Cancer Biomarker Centre, University of Manchester, Manchester, UK; 2 Division of Cancer Sciences, University of Manchester, Manchester, UK; 3 Division of Radiotherapy and Imaging, Institute of Cancer Research, London, UK; 4 The Christie NHS Foundation Trust, Manchester, UK; 5 University College Hospital Macmillan Cancer Centre, Huntley Street, London, UK

**Keywords:** angiogenesis, VEGF inhibitor, response biomarker, Tie2, bile duct cancer

## Abstract

**Background:**

Vascular endothelial growth factor inhibitors (VEGFi) are compromised by a lack of validated biomarkers. Previously we showed that changes in the concentration of plasma Tie2 (pTie2) was a response biomarker for bevacizumab. Here, we investigated whether pTie2 can predict response and progression cross-tumour for generic VEGFi treatment.

**Patients and methods:**

Patients (*n* = 124) with advanced biliary tract cancer (ABC) received cisplatin/gemcitabine with cediranib or placebo (ABC-03 trial). Concentrations of pTie2 were measured longitudinally from before treatment until disease progression. Data from patients with ovarian cancer (*n* = 92, ICON7 trial) and patients with colorectal cancer (CRC) (*n* = 70, Travastin trial) were also included.

**Results:**

Cediranib-treated ABC patients were deconvoluted into distinct groups where in one group pTie2 trajectories resembled those seen in placebo-treated patients and in another pTie2 significantly reduced (*t*-test *P* = 2.7 × 10^−14^). Using the 95% confidence interval for these two groups, we defined a vascular complete response (vCR) as a 24% reduction in pTie2 within 9 weeks; vascular no response (vNR) as a 7% increase in pTie2, and a vascular partial response (between these limits). vCR cediranib-treated patients had significantly improved progression-free survival (8.8 versus 7.5 months, restricted mean ratio 0.73, *P* = 0.012) and overall survival (18.8 versus 12.1 months, hazard ratio 0.49, *P* = 0.02). By integrating data across ovarian cancer, CRC and ABC, we show that (i) patients with vNR do not benefit from VEGFi and (ii) Tie2-defined vascular progression occurs sufficiently in advance of radiological progressive disease that changes in treatment could be offered to prevent clinical deterioration.

**Conclusion:**

pTie2 is the first cross-tumour, generic VEGFi, vascular response biomarker to guide optimum use of VEGFi in clinical practice.

## Highlights

• We identify pTie2 as the first cross-tumour, generic VEGFi, vascular response biomarker.
• A vascular response is defined as at least a 25% reduction in Tie2 within 9 weeks of starting a VEGFi.
• Biliary tract cancer patients attaining a vascular response have improved progression-free and overall survival from a VEGFi.
• Tie2-defined vascular response is valid across both principal classes of VEGFi.
• pTie2 helps predict tumour progression 6 weeks earlier than radiological computed tomography in 61% of patients.

## Introduction

Anti-angiogenic vascular endothelial growth factor pathway inhibitors (VEGFi) have numerous indications in oncology.^1^ In multiple tumour types, addition of VEGFi to conventional cytotoxic chemotherapy regimens improves progression-free survival (PFS) and/or overall survival (OS). Yet, the benefit of modulating tumour vasculature remains modest because we can neither select the patients most likely to benefit nor stop ineffective treatment at an early stage when treatment switching could prove beneficial.^1^

Recent studies have led to new indications for VEGFi including (i) use and re-use effectively in ovarian^2^ and colorectal^3^ cancer (CRC), (ii) co-administration with poly (ADP-ribose) polymerase inhibitor^4^^,^^5^ and (iii) combination with immune checkpoint inhibitors.^6^^,^^7^ Taken together, classical usage of VEGFi coupled with these new indications increases the need for biomarkers to guide use of these effective but somewhat toxic and expensive drugs as effectively as possible.

In previous studies in ovarian^8^ cancer and CRC,^9^ where patients were treated with conventional cytotoxic chemotherapy supplemented by the anti-VEGF antibody, bevacizumab, we showed that plasma Tie2 (pTie2) was a response and progression biomarker for bevacizumab, Tie2 being the endothelial receptor for angiopoietin (Ang) 1 and 2, which promotes angiogenesis through Ang2–Tie2 and VEGF–VEGF receptor 2 axes. We also identified the additive benefit of modelling circulating biomarkers reporting behaviour of both epithelial (cytokeratin 18, CK18) and vascular (pTie2) cells together to predict progressive disease (PD).

Here, we advance the concept of vascular response and develop criteria to define vascular complete response (vCR), vascular no response (vNR) and vascular partial response (vPR). Using data from ABC-03 clinical trial,^10^ we intend to identify patients who are benefited from VEGFi cediranib using the pTie2-defined vascular response criteria, despite at a population level the trial showed no statistically significant benefit from cediranib with respect to PFS or OS. With a further cancer setting, advanced biliary tract cancer (ABC), and an alternative principal class of VEGFi, the small molecule receptor tyrosine kinase inhibitors (RTKi), we systematically evaluated the performance of pTie2 and investigated if pTie2 is a generic response and progression biomarker for VEGFi.

## Patients and methods

### The ABC-03 trial

ABC-03^10^ was a randomised phase II trial in which 124 patients with ABC (cholangiocarcinoma, gallbladder cancer and ampullary cancer) were prescribed cisplatin 25 mg/m^2^ and gemcitabine 1000 mg/m^2^ on days 1 and 8 of a 3-weekly regimen, for up to eight cycles. Patients were randomly allocated (1 : 1) to receive cediranib 20 mg or placebo until PD occurred. The trial results were not statistically significant with PFS intervals of 8.0 months in the cediranib arm and 7.4 months in the placebo group [hazard ratio (HR) 0.93, *P* = 0.72].

Blood samples for biomarker analysis were taken twice before treatment, on the first day of treatment cycles 2-8, at the end of chemotherapy and 1 month after chemotherapy was completed. EDTA-anti-coagulated blood was analysed in a validated multiplex enzyme-linked immunoassay platform (Aushon BioSystems, Billerica, MA), at standards consistent with Good Clinical Practice at the Cancer Research UK Manchester Institute Cancer Biomarker Centre (Manchester, UK).^11^

### Ovarian and colorectal cancer datasets

In addition to data from biliary tract cancer, data from ovarian^8^ cancer (ICON7 trial, 92 patients) and CRC^9^ (Travastin trial, 70 patients) were included to investigate the concept of vascular response and vascular progression. We aimed to verify pTie2 as a pan-tumour, cross-VEGFi response biomarker. The datasets are summarised in Supplementary Table S1, available at https://doi.org/10.1016/j.esmoop.2022.100417.

### Defining cediranib-induced vascular complete response, partial response and no response

**Figure 1:**
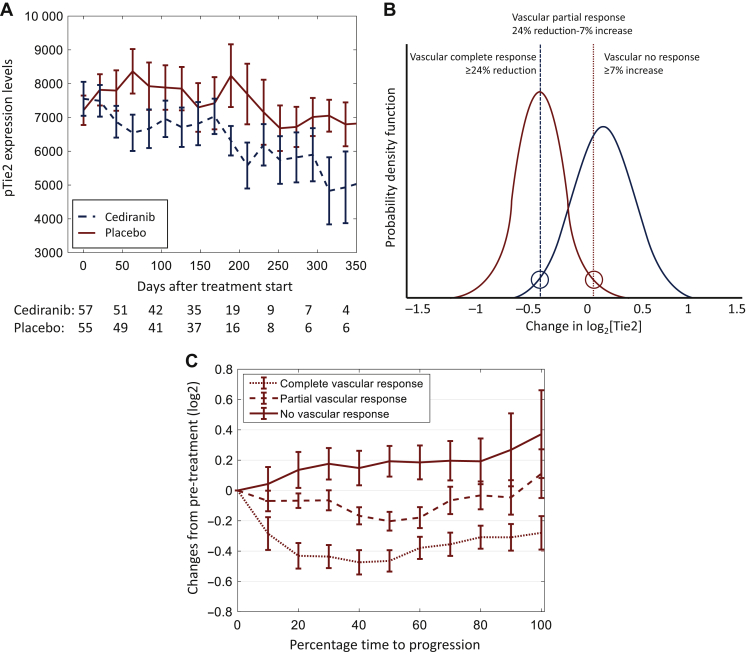
**Plasma Tie2trajectories in ABC-03.** (A) Trajectories of pTie2 according to treatment arms. Trajectories of pTie2 were plotted by treatment arms. The *x*-axis represents the time from the start of treatment. Cediranib arm is presented in a dashed line while the placebo arm in a solid line. pTie2 levels in the cediranib arm were consistently lower than those in the standard arm but did not reach statistical significance at any time point (*P* > 0.15). (B) Deconvolution of cediranib-treated patients into pTie2-defined vascular response groups. Changes in pTie2 during the first three cycles of vascular endothelial growth factor inhibitor (VEGFi) treatment were deconvoluted into two Gaussian distributions: one resembling cytotoxic chemotherapy/placebo-treated patients (blue curve, right), which exhibited moderately elevated pTie2, and one that had a significant reduction in pTie2 concentration (red curve, left; *t*-test versus blue *P* = 2.7 × 10^−14^). The 95% confidence interval (CI) of the blue curve equated with a 24% reduction (left hand circle) in pTie2 such that any patient with >24% reduction was deemed to have had a vascular complete response (vCR). Similarly, patients with a >95% CI increase in pTie2 [red curve, red circle (right)] were defined as vascular no response (vNR). Patients whose pTie2 lay between these two thresholds were deemed to have a vascular partial response (vPR). (C) Plasma Tie2 changes during treatment in patients with Tie2-defined vascular response. Patients were classified as having Tie2-defined vCR (dotted line: at least a 24% reduction in plasma Tie2) or vPR (dashed line: between 24% reduction and 7% increase) or vNR (solid line: at least 7% increase). The *x*-axis represents the interval from the start of treatment to the point of developing progressive disease in percentage. The median progression-free survival (PFS) intervals for those with vCR 263 days, vPR 206 days and vNR 238 days. The median overall survival (OS) intervals were vCR 563 days, vPR 318 days and vNR 482 days.

We aimed to deconvolute patients into different response groups defined by pTie2 changes during treatment. The key hypothesis was that cediranib-treated patients who did not respond to VEGFi had pTie2 trajectories that resembled those seen in patients treated with cytotoxic chemotherapy alone (Figure 1A). Through application of a log scale, a Gaussian mixture model deconvoluted pTie2 changes following VEGFi into two distribution curves: one where pTie2 largely did not change, resembling placebo-treated patients, and the other where pTie2 reduction reflected effective VEGFi treatment (Figure 1B). Three different response groups, vCR, vPR and vNR, were defined using the 95% confidence intervals (CIs) of the two deconvoluted distributions.

### Statistical analysis

The association between pre-treatment pTie2 and patient clinical characteristics was assessed using chi-square tests after dichotomising continuous variables by their median values. The difference in survival (PFS/OS) between patients with vCR and other groups was evaluated using multivariable Cox proportional hazards regression analysis or restricted mean survival if evidence of non-proportional hazards was found. The HR or restricted mean survival time (RMST) was reported, respectively. Established clinical prognostic factors^10^ were adjusted for in these analyses. The analysis follows the Reporting Recommendations for Tumor Marker Prognostic Studies (REMARK) guidelines.^12^

### Defining cediranib-related vascular progression

In ovarian^8^ cancer and CRC,^9^ elevation of pTie2, in patients who had previously attained a vCR, equated with tumour vascular progression, an indication of tumour escaping from VEGF inhibitor control. Using the same Bayesian Markov chain Monte Carlo modelling approach, we investigated rules to define vascular progressive disease (vPD) in cediranib-treated ABC patients. With the hypothesis that vPD is a surrogate of PD, we investigate whether pTie2-defined vPD can be used to predict RECIST-defined radiological progression, and whether combined with the epithelial antigen, CK18, would improve prediction of PD in ABC. The modelling approach has been introduced in previous publication,^8^^,^^9^ and a brief introduction can be found in the Supplementary Appendix, available at https://doi.org/10.1016/j.esmoop.2022.100417.

### Comparison of pTie2 as a vascular biomarker for response and progression across different cancer types and VEGFi

Our previous studies of ovarian^8^ cancer and CRC^9^ showed that bevacizumab-induced reductions and subsequent elevations in pTie2 qualified as vascular responses and vascular progression events, respectively, in both tumour types. The data presented here in ABC, which reflect a different tumour type treated with an alternative class of VEGFi, validate our prior findings with bevacizumab. We therefore integrated the biomarker data predicting disease progression across the three cancer types (ovarian cancer, CRC and biliary tract cancer) to investigate pTie2 performance as pan-tumour, cross-VEGFi biomarker data for treatment response and tumour progression.

All analyses were carried out using Matlab R2019a (MathWorks Inc, Natick, MA) or R version 4.0.^13^

## Results

### ABC patients with Tie2-defined complete vascular response have improved PFS and OS

In ABC-03, 124 patients with ABC were randomly allocated to receive cisplatin and gemcitabine with cediranib or placebo. Pre-treatment pTie2 concentrations were independent of patients’ clinical characteristics (Supplementary Table S2A, available at https://doi.org/10.1016/j.esmoop.2022.100417). Changes in pTie2 concentration according to the allocated arm of treatment are shown in Figure 1A, where pTie2 levels reduced and were consistently lower in the cediranib arm. Over the first 9 weeks (three treatment cycles), patients who received cytotoxic chemotherapy with placebo manifested an average increase in pTie2 of 12% (0.16 ± 0.28 in log_2_ scale). In contrast, plasma CK18 (pCK18), a biomarker known to represent tumour volume, reduced in both arms during treatment as reported in the original biomarker report of ABC-03.^14^

The distribution of pTie2 changes in the placebo arm helped deconvolute those from the cediranib-treated patients into two groups: one in which pTie2 changes resembled those seen in placebo-treated patients and another where there were significant drug-induced reductions in pTie2 (*t*-test *P* = 2.7 × 10^−14^, Figure 1B). Using the 95% CIs of these distributions to define response groups, patients who had at least a 24% reduction in pTie2 over 9 weeks of treatment were deemed to have had a vCR. Patients whose plasma pTie2 concentrations increased by 7% or more had vNR, while the rest had a vPR. The pTie2 trajectories of each vascular response group are shown in Figure 1C. Patients with performance status (PS) of 0 were more likely to achieve vPR/vNR (*P* = 0.04), although this was observed in a small patient cohort (Supplementary Table S2A and B, available at https://doi.org/10.1016/j.esmoop.2022.100417). Otherwise, vascular response was independent of patients’ pre-treatment clinical characteristics. Interestingly, the vascular response status was also independent of changes in pCK18, which reflected tumour volumetric response following treatment (Supplementary Table S2C, available at https://doi.org/10.1016/j.esmoop.2022.100417).

**Figure 2:**
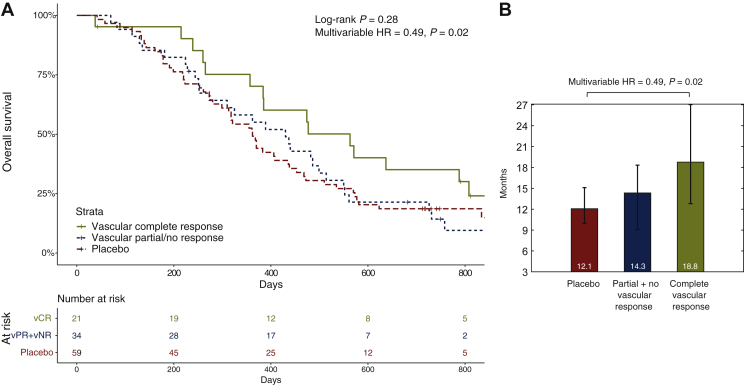
**Plasma Tie2-defined cediranib-induced vascular complete responseis associated with improved overall survivalin ABC-03.** (A) Survival of patients grouped by vascular response status. OS was plotted for patients treated with cisplatin, gemcitabine and cediranib (complete vascular responders, partial + no vascular responders) or placebo. The number of patients at risk at each time point is shown below the Kaplan–Meier curves using the same colour scheme. OS differences were assessed using log-rank tests (*P* = 0.28) and are interrogated in multivariable survival analysis in Figure 2B. (B) Patients with vCR have significantly improved OS. Complete vascular responders to cediranib (*n* = 21) showed improved OS compared to partial/no vascular responders (*n* = 34) and placebo-treated patients (*n* = 59). Patients with vCR had a 6.7-month OS benefit over placebo, equating to a hazard ratio (HR) of 0.49 which was statistically significant in multivariable analysis after adjusting for clinical prognostic factors (*P* = 0.02). vPR, vascular partial response.

The original analysis of ABC-03^10^ did not detect a cediranib-associated improvement in PFS or OS. However, we hypothesised that this had occurred because of an inability to separate those who had a vascular response from those who had not. Having defined a vCR as a reduction in pTie2 of ≥24% in the first 9 weeks, we observed that vCR (21 patients, 38%) was associated with a median OS of 18.8 months, as opposed to 14.3 months and 12.1 months for patients with vPR/vNR (34 patients, 62%) and placebo (59 patients, Figure 2), respectively. A multivariable Cox regression analysis adjusted for prognostic clinical factors confirmed that vCR predicted OS benefit in the cediranib arm but not in the placebo arm (Supplementary Table S3A, available at https://doi.org/10.1016/j.esmoop.2022.100417, *P* < 0.001). Further analysis indicated that patients with vCR from cediranib had a significantly improved OS compared to placebo patients (6.7 months, multivariable *P* = 0.023, HR 0.49, 95% CI 1.102-3.752, Supplementary Table S3B, available at https://doi.org/10.1016/j.esmoop.2022.100417). Similarly, patients with vCR had an improved PFS when compared with those who had vPR/vNR or placebo-treated patients (Supplementary Figure S1, available at https://doi.org/10.1016/j.esmoop.2022.100417, 8.8, 7.5 and 8.0 months, respectively). Non-proportional hazards in PFS were observed mandating a restricted mean survival analysis, which demonstrated a significantly improved RMST ratio of 0.73 (*P* = 0.012, Supplementary Table S4, available at https://doi.org/10.1016/j.esmoop.2022.100417) in favour of patients with complete vascular response against vPR/vNR, and an RMST ratio of 0.77 (*P* = 0.001) against placebo.

### pTie2 is the first cross-tumour, generic VEGFi, vascular response biomarker

Evaluating the concept of vCR in other tumour types, in ovarian cancer, an elevation in pTie2 of 0.08 ± 0.26 (log_2_ scale) was observed in patients treated with cytotoxic chemotherapy over the first three treatment cycles. Similarly, in CRC, the increase was 0.10 ± 0.24 (log_2_ scale), indicating that pTie2 expression levels change consistently across cancer types when treated with cytotoxic chemotherapy in the absence of a VEGFi. Thus, the thresholds for defining vCR were consistent regardless of cancer types, small molecule- or antibody-based VEGFi, allowing us to define vCR as being a decrease in pTie2 of ∼24%-30% across all three tumour types within 9 weeks of starting treatment.

In CRC, a 30% reduction or more from pre-treatment pTie2 concentrations in 9 weeks after treatment started was considered a vCR while 4% increase or more was considered vNR. There were too few early data points in the ovarian cancer dataset to allow this sub-classification of response. The proportion of patients falling into each vascular response group and the corresponding treatment benefit in PFS and OS are listed in Tables 1 and 2. Compared to patients with ABC, a larger proportion of the CRC patients were deemed to have had a vCR (47% versus 38%). Coupled with the toxicity-associated early withdrawal of treatment in some patients, the lower proportion of cediranib-treated ABC patients who benefited from the VEGFi, in contrast to patients with CRC, explains the negative trial results from ABC-03.

**Table 1:** Vascular response rates in ABC and CRC

	vCR, %	vPR, %	vNR, %
ABC cediranib	38	36	25
CRC bevacizumab	47	29	24

Distribution of ABC and CRC patients, treated with cytotoxic chemotherapy and a VEGFi, cediranib (ABC) and bevacizumab (CRC), into those with a vCR, vPR and vNR.

ABC, advanced biliary tract cancer; CRC, colorectal cancer; vCR, vascular complete response; VEGFi, vascular endothelial growth factor inhibitor; vNR, vascular no response; vPR, vascular partial response.

**Table 2:** Risk of progression/death in patients with different vascular response

	vCR		vPR		vNR	
	PFS, HR^a^	OS, HR^a^	PFS, HR^a^	OS, HR^a^	PFS, HR^a^	OS, HR^a^
ABC cediranib	1	1	1.71	3.09	1.71	1.15
CRC bevacizumab	1	1	0.99	0.80	1.40	1.00

Hazard ratios (HRs) for developing progressive disease (PFS) or dying from disease (OS) are shown according to the disease considered (ABC or CRC) and the association with vascular response category (vCR, vPR or vNR). Each box contains the HR presented in the format PFS and OS. HR values >1 represent an increased likelihood of developing progressive disease or dying from the disease, respectively.

ABC, advanced biliary tract cancer; CRC, colorectal cancer; OS, overall survival; PFS, progression-free survival; vCR, vascular complete response; vNR, vascular no response; vPR, vascular partial response.

a Higher HR indicates higher risk of progression or death, which typically translate to reduced PFS or OS.

It should be noted that the thresholds for vascular response were mathematically derived and by definition vPR comprises patients with varied magnitude of treatment benefit from VEGFi. Indeed, the clinical heterogeneity was demonstrated in that the treatment benefit in vPR patients was small in ABC-03 but superior in patients with CRC (Table 2). Thus, different cancers will have varied optimum cut-offs for clinical decision making. In ABC, the optimum cut-off was identified as 24% reduction from pre-treatment pTie2 corresponding exactly with the mathematically derived vCR threshold. In CRC patients, instead, a reduction of ≥10%, which lay in the middle of the vPR range (30% reduction to 4% increase) identified a patient cohort with a PFS benefit of HR = 0.52 (*P* = 0.01, Supplementary Figure S2, available at https://doi.org/10.1016/j.esmoop.2022.100417).

### Vascular biomarker pTie2 and epithelial biomarker pCK18 jointly predict progressive disease before radiological detection

**Figure 3:**
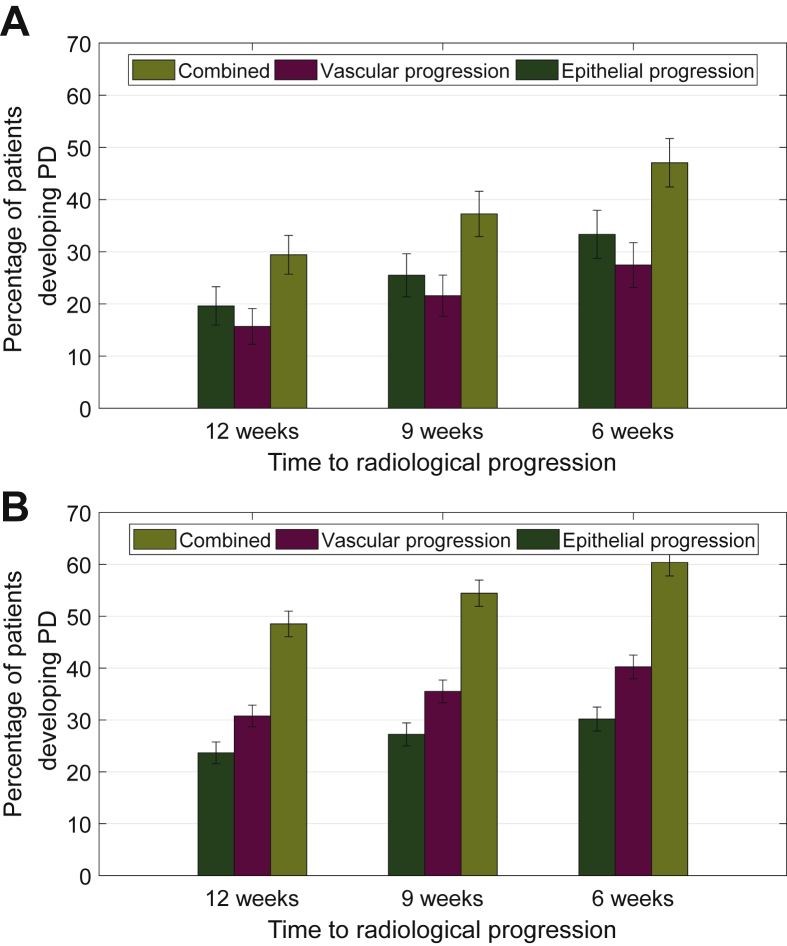
**Tie2-defined vascular response and progression in advanced biliary tract cancer (ABC) and in three tumour types.** (A) Modelling vascular and epithelial biomarker data together for patients with ABC, treated with cediranib. Co-modelling of Tie2 vascular biomarker-defined progression, with CK18 epithelial biomarker-defined progression to form a combination of vascular and epithelial biomarkers allowed detection of progression in up to 47% of patients in ABC-03, 6 weeks before RECIST progression was detected. The combined biomarkers detected significantly more patients with progressive disease (PD) than epithelial or vascular biomarkers did alone (*P* < 0.001). (B) Modelling vascular and epithelial biomarker data together across three tumour types. Using the same colour scheme as Figure 3A, when data from ovarian (48 patients, CA125 was the epithelial marker), colorectal (70 patients, CK18 was the epithelial marker) and biliary tract cancers were summated, 61% of patients had PD that was detectable 6 weeks before RECIST-defined radiological progression. The combined biomarker detected significantly more patients than epithelial or vascular biomarker alone (*P* < 0.001). This is a highly significant effect that allows PD to be detected before radiological progression with enough time that a new treatment option could be introduced, thereby preventing radiological progression.

Using the same approach as we applied in CRC,^9^ an increase in pTie2 of 40% from nadir defined vPD in ABC. For pCK18, a 50% increase from nadir was determined to predict PD. Co-modelling of pTie2 and pCK18 biomarker data together improved prediction of radiological PD when compared with the predictive value of either circulating biomarker alone (*P* < 0.001, Figure 3A). Thus, 6 weeks before the detection of radiological progression, we were able to predict PD in 47% of ABC patients using circulating biomarkers.

Integrating data across ovarian cancer, CRC and ABC, we achieved a dataset comprising 286 patients, where vascular biomarker pTie2 and epithelial biomarkers CA125 or pCK18 were measured longitudinally. We observed significantly improved prediction of radiological PD (*P* < 0.001) by jointly using epithelial and vascular biomarkers from 12 to 6 weeks before radiological progression (Figure 3B). Collectively, PD can be anticipated in 61% of patients, 6 weeks before radiological progression, affording clinicians time to change treatment potentially before clinical/radiological progression occurs. The data shown in Figure 3 increase clinical confidence in the concept of pTie2-defined vascular progression as there was statistically significant additivity between the epithelial and vascular biomarker data.

## Discussion

In this work, we showed that pTie2 is the first cross-tumour, vascular response biomarker for generic VEGFi. In three different tumour types, where patients were treated with two different classes of VEGFi, pTie2 can define vascular response and vascular progression based on its changes after treatment. We found that pTie2 concentrations and vascular response status are independent of most clinical features, despite that patients with Eastern Cooperative Oncology Group PS of 0 are less likely to be vCR. However, this needs further verification due to small sample size and multiple comparison.

This study is the first demonstration of the clinical value of a vascular response biomarker in the setting of VEGF RTKi-treated patients, and importantly, it shows that there is a group of patients with ABC who gain clinically significant improvements in PFS and OS from cediranib. Clinical qualification of the pTie2 test is now warranted given its potential during the first few weeks of treatment to inform decision making by oncologists and their patients on whether to continue taking cediranib. The significant clinical benefit in vCR patients should prompt return to relatively VEGFi-resistant tumour types such as breast cancer, to determine the benefit in the subgroup of patients who attain a pTie2-defined vCR. Further work is required to understand the vPR group as this cohort did not benefit from cediranib in ABC but did benefit from bevacizumab in a second dataset of patients with CRC^9^ (Tables 1 and 2, Figure 2, Supplementary Figures S1 and S2, available at https://doi.org/10.1016/j.esmoop.2022.100417). This may be related to the mixture of vascular responses contained in the vPR group and the impact of VEGFi-related toxicity on treatment withdrawal. As the definitions of vPR are mathematically derived, and we have shown that a better categorisation can be derived within the vPR for different cancer types, further clinical studies are warranted for the identification of an optimised threshold for decision-making purposes, e.g. our ongoing clinical trial, VALTIVE1.^15^

The differential vascular response rate between different tumour types has significant implications for cancers that were previously deemed not to benefit from VEGFi. For instance, early research showed that VEGFi were clinically active in some patients with breast cancer,^16^ yet ultimately approval for VEGFi was withdrawn by the Food and Drug Administration for this indication.^17^ This decision was in part related to insufficient activity and therefore it is fair to assume that in diseases where some patients but not the overall population benefit from VEGFi, e.g. breast or prostate cancer, the proportion of patients with VEGFi-responsive disease is likely to be ≤50%. Assuming that patients whose disease responds to VEGFi have HRs for progression of ∼0.5 or 0.6, we can now outline phase II biomarker studies that would test the hypothesis that pTie2 identifies the patients who benefit from VEGFi. For example, if we wanted to test the hypothesis in breast cancer that there was a 40% vascular response rate and an HR for PFS of 0.5 between vascular responders and non-responders, using a power of 80% and an error of 0.05, we would need to treat 85 patients with a VEGFi-containing regimen; if we propose that there is a 30% vascular response rate and an anticipated HR of 0.5, this would require 98 patients. The cytotoxic regimens used in our studies so far have not impacted pTie2 readout, so it would be reasonable to study any conventional cytotoxic–VEGFi combination. Patients would have serial measurements of pTie2 allowing the group to be split into vascular responders and non-responders to test this hypothesis where a positive result could lead to licensing of the VEGFi based on Tie2-defined vascular response.

We have discovered and now validated in three cancer types the concept of additivity between epithelial and vascular circulating biomarkers with respect to predicting PD. In addition, we observed that pTie2-defined vascular response status is independent from tumour volumetric changes as reflected by pCK18. Conceptually, this is important as it provides increased confidence of the interdependency of two notional tissue compartments in tumour growth reported with a surrogate liquid biopsy. Our data support testing the hypothesis that vascular and epithelial progression should be managed independently based on corresponding biomarkers, and that patients who develop vascular progression could be prevented from developing clinical/radiological progression through the administration of drugs that overcome resistance to VEGFi. Given that macrophages can promote angiogenesis^18^ and have been implicated in mediating resistance to VEGFi,^19^ that upon vascular progression we are seeing an increase in pTie2, which is expressed on a subset of macrophages and that preclinical data show that colony-stimulating factor 1 receptor antagonists overcome acquired resistance to VEGFi, *in vivo*,^20^ it would be pertinent to evaluate macrophage inhibitors as potential agents to reverse pTie2-defined vascular progression in patients receiving VEGFi.

Extrapolating further, as pTie2 and pCK18 trajectories predicted progression in 61% of patients with ovarian cancer, CRC or biliary tract cancer before radiological progression, we reason that additional tissue-specific circulating biomarkers, e.g. reporting on the immune system, could be identified that further increase our ability to anticipate PD and by changing treatments at a sufficiently early stage, avoid the development of clinical and/or radiological progression. Such an approach would change cancer from a relapsing and remitting condition to a much more stable disease through the application of lineage-specific biomarkers in a ‘multi-tissue compartment model’ of cancer management.

## Supplementary data

Supplementary Appendix

Supplementary Figure S1A

Supplementary Figure S1B

Supplementary Figure S2A

Supplementary Figure S2B
