# Supplementary material for: Plasma Tie2 trajectories identify vascular response criteria for VEGF inhibitors across advanced biliary tract, colorectal and ovarian cancers
Source: ESMO Open. 2022 Mar 10;7(2):100417. doi: 10.1016/j.esmoop.2022.100417 (PMC9058891; doi:10.1016/j.esmoop.2022.100417)
Supplement: Supplementary Appendix [file mmc1.docx]

**Appendix**

In this appendix a brief introduction is given on how to use a Bayesian MCMC modelling approach to model pTie2 trajectories and how to use the developed model to infer clinical rules that predict tumour progression.

**Modelling pTie2 longitudinal trajectories**

Trajectories of pTie2 were modelled using a Bayesian hierarchical modelling approach. The approach aimed to develop probability models that define joint probability distribution for all observed and unobserved data. A piecewise-linear relationship between pTie2 concentration and treatment time was assumed where an inflection point separated the decreasing part of pTie2 trajectory from the subsequent increasing component. The inflection point indicates the development of VEGFi resistance and therefore as the earliest sign of tumour progression.

The Bayesian hierarchical model was set up based on a piecewise linear relationship between biomarker quantity (concentration for circulating biomarkers; various units for imaging biomarkers) and treatment time (t), parameterized as follows:

$$C_{biomarker}\left( t \right)=\alpha+S\left( t_{inflection}-t \right)\beta t+S\left( t-t_{inflection} \right)\left( \beta t_{inflection}+\gamma\left( t-t_{inflection} \right) \right)+\varepsilon$$

where C represents the log2 transformed concentration of pTie2, α is the pre-treatment concentration of the biomarker, t_inflection_ is the inflection point of pTie2 trajectory, β is the slope before the inflection point, γ is the slope after the inflection and ε is a random error. S is an indicator function where

$$S\left( x \right)=\left\{ \begin{aligned} 1 if x>0 \\ 0 if x\leq0 \end{aligned} \right.$$

The parameters were assigned the following prior distributions to follow the structure of a Bayesian hierarchical model:

$$\alpha\sim N\left( \mu_{\alpha},\sigma_{\alpha}^{2} \right)$$

$$\beta\sim\alpha\times\left( \beta_{1}+{E_{1}\times\beta}_{2} \right)/\mu_{\alpha}$$

$$\beta_{1}\sim N\left( \mu_{\beta1},\sigma_{\beta1}^{2} \right)$$

$$\beta_{2}\sim N\left( \mu_{\beta2},\sigma_{\beta2}^{2} \right)$$

$$E_{1}\sim B\left( 1, p_{1} \right)$$

$$\gamma\sim\alpha\times\left( \gamma_{1}+{E_{2}\times\gamma}_{2} \right)$$

$$\gamma_{1}\sim N\left( \mu_{\gamma1},\sigma_{\gamma1}^{2} \right)$$

$$\gamma_{2}\sim N\left( \mu_{\gamma2},\sigma_{\gamma2}^{2} \right)$$

$$E_{2}\sim B\left( 1, p_{2} \right)$$

$$\varepsilon\sim C\times N\left( 0,\sigma_{\varepsilon}^{2} \right)$$

$$t_{inflection}\sim U(21, PFS-21)$$

Specifically, α follows a log normal distribution, β is dependent on α and both β, γ are modelled to follow a combination of normal distribution that controlled by E1 and E2 (Bernoulli distributions). The reason for setting β and γ as above is to accommodate the fact that Tie2 trajectories can be distinct in responding and non-responding patients. ε can be considered as technical variation and is therefore modelled to be dependent on biomarker quantity. The inflection is uniformly distributed between 21 days after treatment starts and 21 days before diagnosis of progression.

The posterior distributions of the parameters were determined using an MCMC approach as implemented in Winbugs 1.4. For each model, three Markov chains were trained at a same time to ensure best coverage. They were updated for 50,000 iterations or until sufficient evidence of model convergence was observed, whichever occurred later. A converged model will be updated for a further 100,000 iterations to estimate posterior distribution of each model parameter. According to our observations, convergence was achieved typically within 20,000 update iterations.

**Predicting tumour progression using pTie2 longitudinal model**

The Bayesian hierarchical model, once developed, enabled us to address two questions. Firstly, it allowed determination of the inflection point, i.e., considered as a sign of VEGFi resistance and the first sign of progressive disease. Secondly, it can estimate pTie2 concentrations at a given time during treatment for a given patient. For example, the model enabled inference of the concentration of pTie2 on a pseudo trial carried out on the same cohort of patients but on different sample collection schemes. This inference process was different from the concept of “prediction”, as it did not generate data for a “new patient” based on an “existing patient”. Instead, such estimation resembles interpolating values for missing data points utilising information from individual patients and the whole population. Pseudo-trial data can be used to determine optimum rules for biomarkers to predict tumour progression, which will be described in detail below.

We intended to develop rules on how to use the selected biomarkers in clinic, that is, how to provide early predictions of tumour progression by monitoring the biomarkers sequentially during treatment. Prediction of tumour progression was considered if elevation of pTie2 with respect to its recorded nadir point exceeds a designated "alarm" threshold. The optimal threshold can be determined using the following steps:

(1) For any threshold we generated pseudo-trial data for a given biomarker via inference from the Bayesian model at a designated time. In this study a monthly sample collection interval (30 ± 5 days) was applied.

(2) For each patient, examine recorded data sequentially following treatment time. For a given time point record the percentage change of biomarker concentration with respect to nadir concentration was recorded prior to this time point. If the percentage change were larger than the designated threshold value, and the time point was at least 45 days after treatment, we recorded the time point and considered that a prediction of tumour progression had been made.

(3) We repeated steps 1-2 five thousand times to fully sample the model.

(4) Biomarker prediction performance was evaluated by summarising all the recorded prediction times.

The threshold was chosen to predict PD for as many patients as possible, at a time reasonably close to the radiological PD.
