# Supplementary figures and images for: Plasma Tie2 trajectories identify vascular response criteria for VEGF inhibitors across advanced biliary tract, colorectal and ovarian cancers
Source: ESMO Open. 2022 Mar 10;7(2):100417. doi: 10.1016/j.esmoop.2022.100417 (PMC9058891; doi:10.1016/j.esmoop.2022.100417)

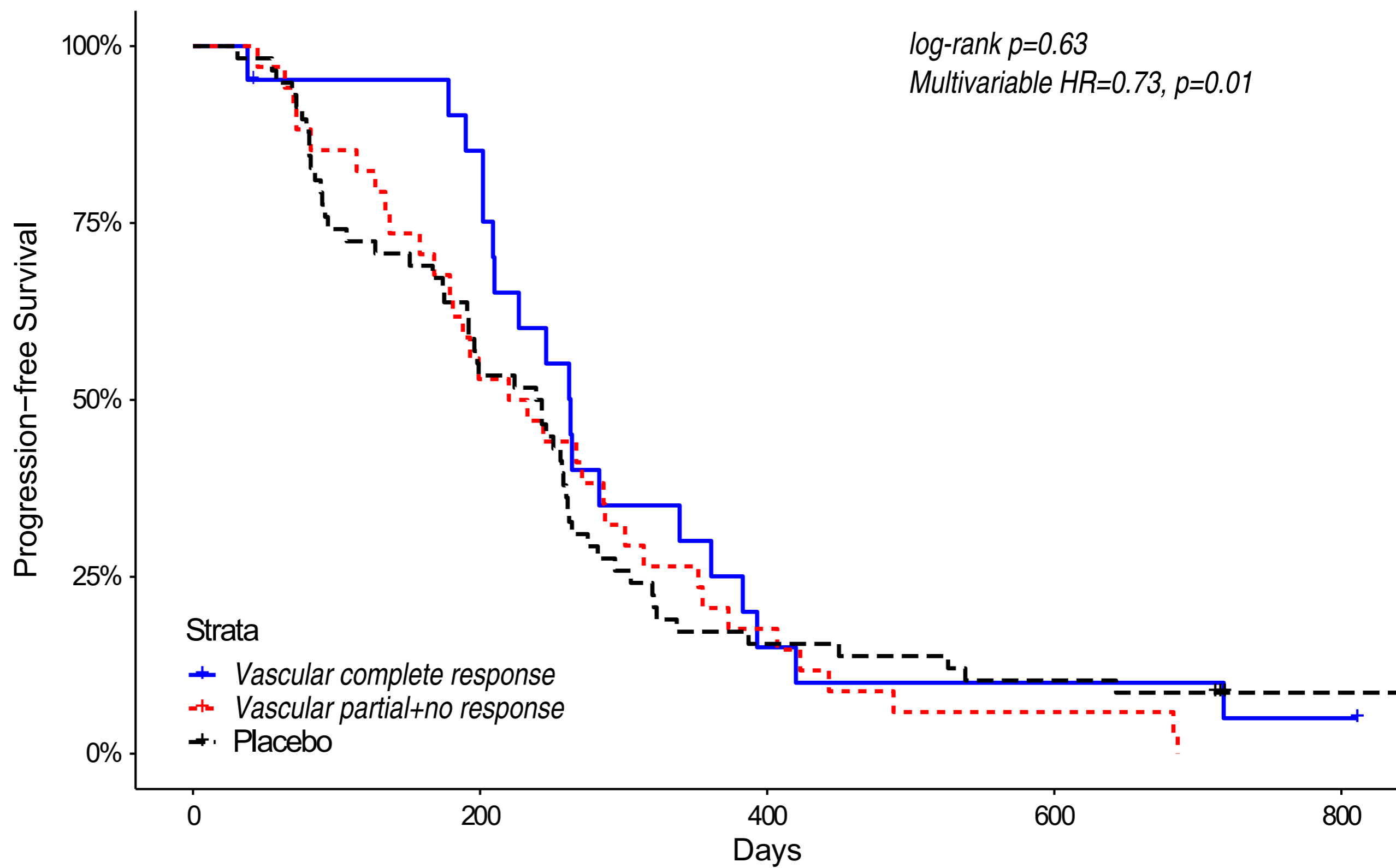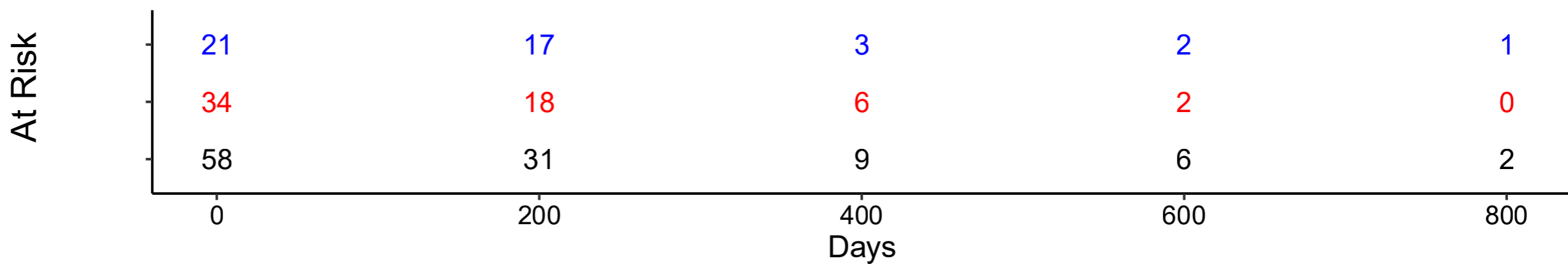

Supplement: Supplementary Figure S1A [file mmc2.pdf]

*Multivariable RMST ratio=0.73, p=0.01*

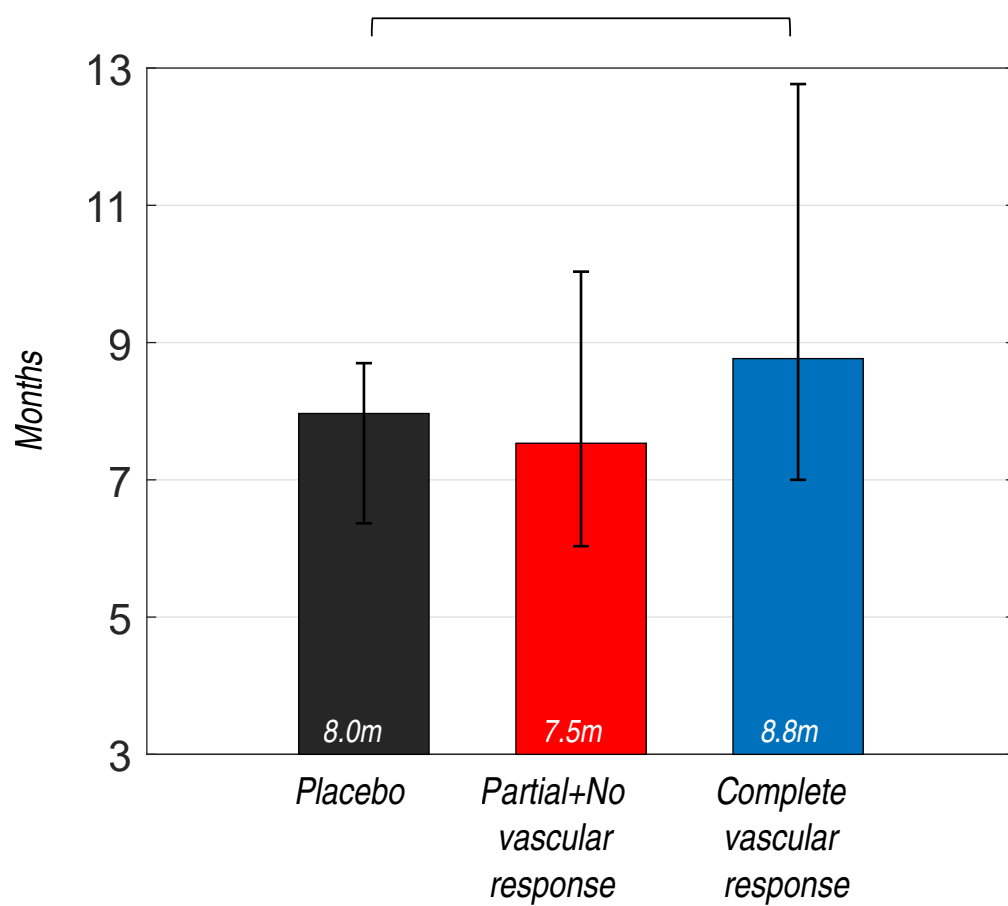

Supplement: Supplementary Figure S1B [file mmc3.pdf]

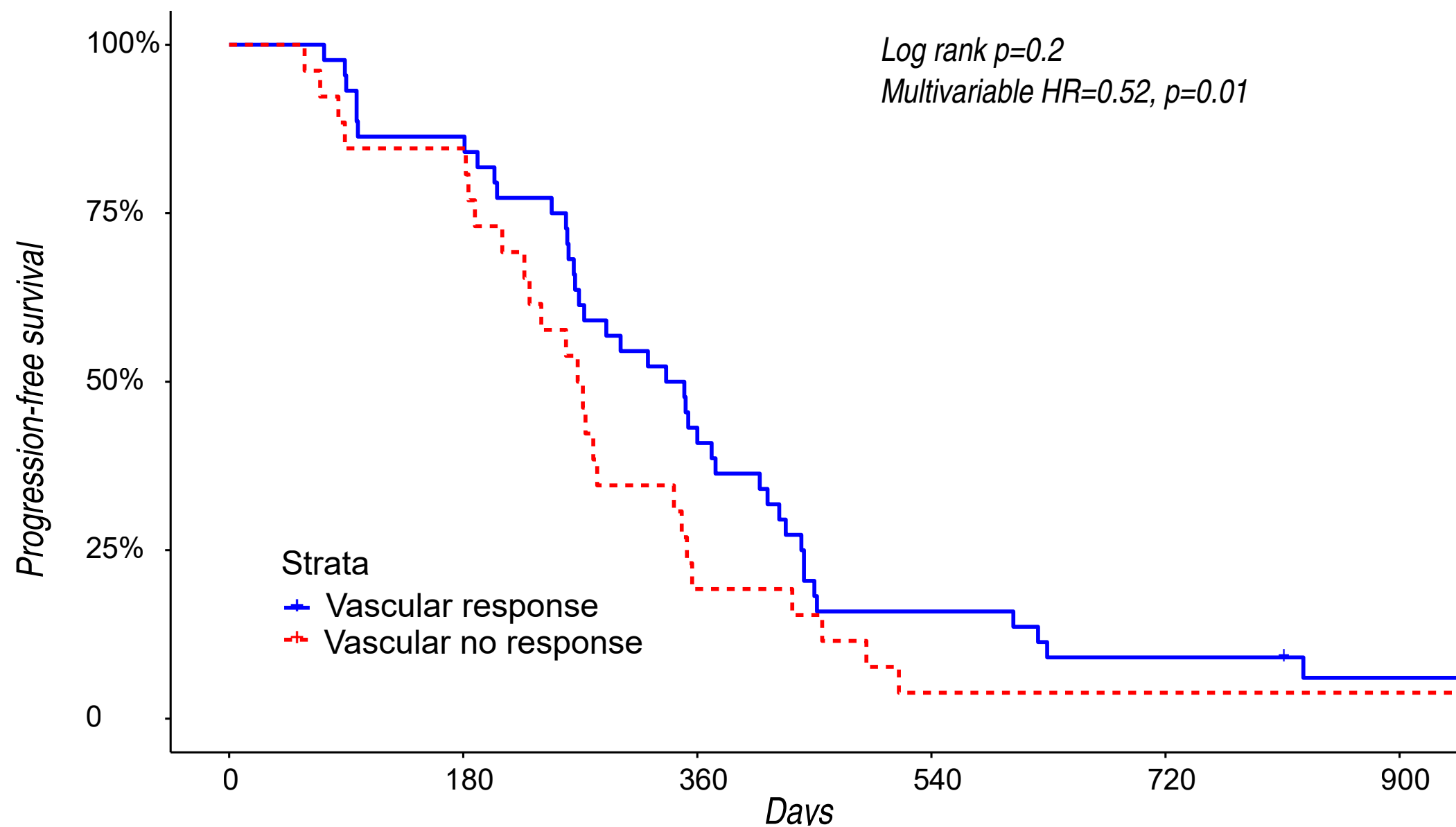

Number at risk

|                      |      |     |     |     |     |     |
|----------------------|------|-----|-----|-----|-----|-----|
| Vascular response    | 44   | 38  | 19  | 7   | 4   | 2   |
| Vascular no response | 26   | 22  | 5   | 1   | 1   | 1   |
|                      | 0    | 180 | 360 | 540 | 720 | 900 |
|                      | Days |     |     |     |     |     |

Supplement: Supplementary Figure S2A [file mmc4.pdf]

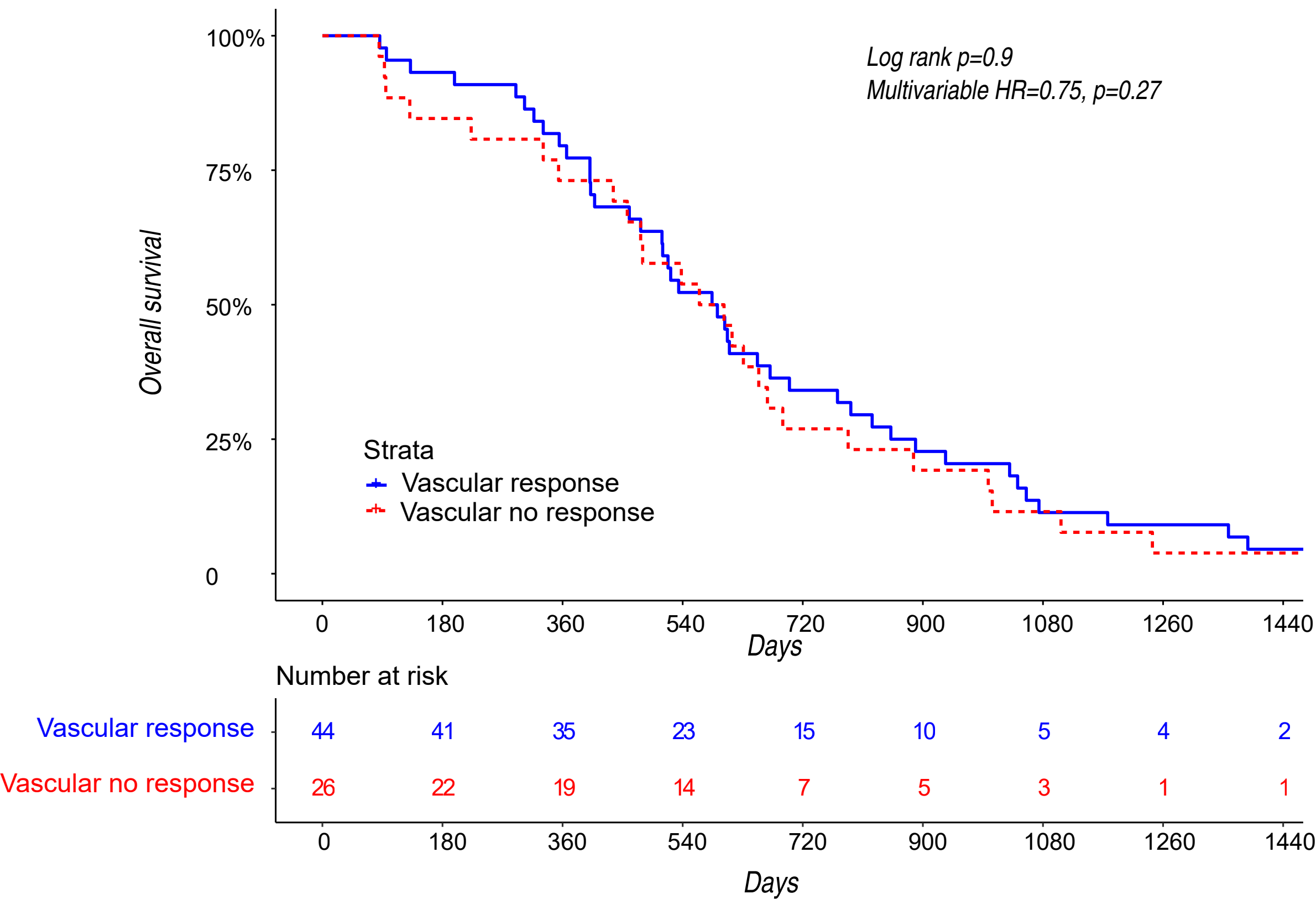

Supplement: Supplementary Figure S2B [file mmc5.pdf]
