# Supplementary material for: Plasma Tie2 trajectories identify vascular response criteria for VEGF inhibitors across advanced biliary tract, colorectal and ovarian cancers
Source: ESMO Open. 2022 Mar 10;7(2):100417. doi: 10.1016/j.esmoop.2022.100417 (PMC9058891; doi:10.1016/j.esmoop.2022.100417)
Supplement: Supplementary Tables S1-S4 [file mmc6.docx]

**SUPPLEMENTARY TABLES**

***Supplementary table 1. Datasets used in the study***

| Dataset | N (total / VEGFi) | Disease | Treatment | Trial setting |
| --- | --- | --- | --- | --- |
| Icon7 | 92 /48 | Ovarian cancer | Chemotherapy +/- bevacizumab | Randomised two arms |
| Travastin | 70 / 70 | Metastatic colorectal cancer | Chemotherapy + bevacizumab | Single arm |
| ABC-03 | 116 / 51 | Advanced biliary tract cancer | Chemotherapy +/- cediranib | Randomised two arms |

**Supplementary table 2. Relationship between clinical characteristics and *vascular response***

***Supplementary table 2a. Association between pre-treatment clinical characteristics and pTie2 defined vascular response***

|  | Age | Gender | Stage | ECOG PS | Histology | Primary site |
| --- | --- | --- | --- | --- | --- | --- |
| **Pre-treatment pTie2 concentration** | 0.166 | 0.516 | 0.828 | 0.506 | 0.123 | 0.696 |
| **Vascular response status** | 0.916 | 0.874 | 0.367 | **0.040** | 0.411 | 0.374 |

P-values from chi-squared were listed. Clinical characteristics are independent from pre-treatment pTie2 levels except from ECOG Performance status. ECOG Performance status demonstrated a strong association with pTie2 defined vascular response status.

***Supplementary table 2b. ECOG performance status break down by vascular response***

|  | PS: ambulatory | PS: fully active |
| --- | --- | --- |
| **Vascular complete response** | 16 | 5 |
| **Vascular partial/no response** | 15 | 19 |

Patients with ECOG Performance status of 0 are more likely to have vascular partial response or no response.

***Supplementary table 2c. Association between changes of tumour volume and pTie2 defined vascular response***

|  | pCK18 changes from pre-treatment to treatment cycle 3 | pCK18 changes from pre-treatment to treatment cycle 6 |
| --- | --- | --- |
| **pTie2 changes from pre-treatment to treatment cycle 3** | p=0.228, r=0.115 | p=0.390, r=0.082 |
| **Vascular response status** | p=0.595 | p=0.999 |

Pearson’s correlation coefficients and p-values were listed to evaluate the association between changes of pTie2 and pCK18 during treatment. P-values from chi-squared tests were listed to evaluate the association between vascular response status and pCK18 changes dichotomised by median. pTie2 is independent of pCK18 on their changes during treatment.

**Supplementary table 3. Overall survival of vascular complete responders in ABC-03**

***Supplementary table 3a. Patients with vascular complete response have significantly improved OS***

| Covariate | Description | P-value | HR | 95% CI |
| --- | --- | --- | --- | --- |
| Vascular response:  Treatment* | Interaction | <0.001 | 0.140 | 0.048-0.412 |
| Vascular response | vPR+vNR vs. vCR | 0.048 | 1.938 | 1.006-3.734 |
| Treatment | Placebo vs. Cediranib | <0.001 | 6.873 | 2.630-17.963 |
| CEA** | Doubling | <0.001 | 1.230 | 1.100-1.375 |
| Ca19-9** | Doubling | 0.068 | 1.053 | 0.996-1.113 |

* Interaction between vascular response and treatment

** log2 transformed

Vascular complete response (vCR) patients had a significant OS benefit in the cediranib arm but not in the placebo arm. The multivariable model was adjusted for CEA and Ca19-9 which were prognostic for OS.

***Supplementary table 3b. Interpretation of OS benefit in patients with vascular complete response***

| Covariate | P-value | HR | 95%CI |
| --- | --- | --- | --- |
| Vascular complete response |  | 1 |  |
| Vascular complete response vs. vascular partial + no response | 0.056 | 0.527 | 0.985-3.653 |
| Vascular complete response vs. placebo | **0.023** | **0.492** | **1.102-3.752** |

The multivariable model listed in supplementary table 3a was interpreted by estimating hazard ratios among vCR, vPR/vNR and placebo.

**Supplementary table 4. Progression-free survival of vascular complete responders in ABC-03**

***Supplementary table 4a. Patients with vascular complete response have significantly improved PFS***

| Covariate | Description | P-value | HR | 95% CI |
| --- | --- | --- | --- | --- |
| Vascular response:  Treatment* | Interaction | <0.001 | 0.080 | 0.025-0.258 |
| Vascular response | vPR+vNR vs. vCR | 0.087 | 1.767 | 0.920-3.395 |
| Treatment | Placebo vs. Cediranib | <0.001 | 8.845 | 3.182-24.588 |
| Metastatic disease | Yes vs. No | 0.023 | 2.099 | 1.106-3.984 |
| ECOG performance status | Full active vs. Ambulatory | <0.001 | 0.385 | 0.234-0.635 |
| CA125** | Doubling | 0.055 | 1.134 | 0.998-1.290 |

* Interaction between vascular response and treatment

** log2 transformed

Vascular complete response (vCR) patients demonstrated a significant PFS benefit in cediranib arm but not in the placebo arm. The multivariable model has adjusted for ECOG PS, metastatic disease status and CA125 which were prognostic for PFS.

***Supplementary table 4b****.* ***Interpretation of PFS benefit in vascular complete response***

| Covariate | P-value | RMST | 95%CI |
| --- | --- | --- | --- |
| Vascular complete response |  | 1 |  |
| Vascular complete response vs. vascular partial+no response | **0.012** | **0.728** | **0.566-0.936** |
| Vascular complete response vs. placebo | 0.001 | 0.773 | 0.700-0.854 |

The multivariable model listed in supplementary table 4a was interpreted by estimating hazard ratios among vCR, vPR/vNR and placebo. Restricted mean survival time (RMST) was used due to non-proportional survival. vCR patients showed significantly extended PFS compared to vPR/vNR or placebo.
