# Supplementary material for: Plasma Tie2 trajectories identify vascular response criteria for VEGF inhibitors across advanced biliary tract, colorectal and ovarian cancers
Source: ESMO Open. 2022 Mar 10;7(2):100417. doi: 10.1016/j.esmoop.2022.100417 (PMC9058891; doi:10.1016/j.esmoop.2022.100417)
Supplement: Supplementary Figures [file mmc7.docx]

**Supplementary Figure 1 Tie2-defined vascular complete responders to cediranib have improved PFS in ABC-03**

**Supplementary Figure 1a Survival of patients grouped by vascular response status** Progression-free survival were plotted for patients treated with cisplatin, gemcitabine and cediranib (blue line, complete vascular responders; red line, partial + no vascular responders) or placebo (black line). The numbers of patients at risk at each time point are shown below the Kaplan-Meier curves using the same colour scheme. The PFS difference was assessed using log-rank tests (*P*=0.63) and was evaluated in multivariable survival analysis in Fig S1b.

**Supplementary Figure 1b. Vascular complete responders have significantly improved PFS than placebo**

Complete vascular responders to cediranib (blue bar, *n*=21) showed improved PFS compared to partial/no vascular responders (red bar, *n*=34) and placebo treated patients (black bar, *n*=59). A restricted mean survival analysis showed that complete vascular responders have an RMST ratio of 0.73 compared to partial/no responders after adjusting for clinical prognostic factors (*P*=0.01).

**Supplementary Figure 2 Improved categorisation of vPR patients in CRC**

An improved threshold can be defined within the range of mathematically derived vPR, leading to a clinically more meaningful categorisation of patients who benefit from VEGFi.
